# Does a cluster always equal a cluster? Geographical variation of cluster populations

**DOI:** 10.1192/pb.bp.113.045237

**Published:** 2014-12

**Authors:** Josephine Morgan

**Affiliations:** 1 Camden and Islington NHS Foundation Trust

## Abstract

**Aims and method** To provide information regarding the extent to which the process of clustering using the mental health clustering tool captures the complexity of patient need across different geographical areas. Investigation was undertaken via a ‘deep dive’ into patient notes, with data collected on patients allocated to cluster 5, 8 or 13 in three different London boroughs.

**Results** There is evidence for within-cluster differences between patients in different London boroughs in terms of various complexity factors. Further findings in relation to accuracy of clustering suggest some area-specific patterns in terms of clustering practice, raising the possibility that clinicians have different scoring thresholds in different areas.

**Clinical implications** Complexity factors can affect resource use and therefore cost of service provision. In the case of a national tariff, providers of care to more complex patients may be placed at greater financial risk. It is therefore likely that some form of tariff adjustments will need to be introduced so as not to disadvantage patients and clinicians practising in areas of greater complexity.

The introduction of payment by results (PbR) in mental health is well under way; for the year 2013/2014, all contracts between commissioners and providers should have been re-based according to a mental health cluster.^1^ For the year 2014/2015, it is intended that progress will continue, with the clusters being used to agree local prices.^2^ The Department of Health’s ultimate aim is the development of national tariffs for clusters, as it is felt that these will support the delivery of more consistent services.^1^ It is therefore of the utmost importance that the characteristics of the mental health clustering tool (MHCT), the tool underpinning the PbR process, are fully understood. Although the MHCT has been through extensive evaluation during its development,^3^ a number of concerns have been raised as it becomes more routinely used. In particular, there are concerns about significant heterogeneity within clusters, given the relatively few clusters available for allocation (20 in total). The concept of ‘complexity factors’ (factors which are not reflected by cluster allocation but are likely to increase the complexity of care required) relates to this. The nature and extent of complexity factors may differ from area to area, affecting local prices and providing a challenge in the development of a national tariff.

The importance of complexity is well recognised in acute care PbR, and this system provides a stark contrast to that proposed for mental health. In the acute care system, around 26 000 codes are used to describe diagnoses and interventions given, which are grouped into over 1500 tariffs.^4^ Some of these tariffs are split to differentiate between patients with and without complications and comorbidities, and some are split to give a higher tariff where best practice has been provided.^1^ As well as these tariff subsets, various post-tariff adjustments are made; for example, length of stay adjustments, specialised service top-up payments, adjustments for extra emergency work done, and a final adjustment called the market forces factor.^1^

In London, concerns about the potential role of complexity factors have been expressed at forums supporting the implementation of PbR locally.^5^ These discussions led to a proposal for a local project exploring cluster complexity across areas with populations with differing socioeconomic profiles.

## Aim

The specific question this project aimed to address was: ‘When comparing cluster X patients in borough A with cluster X patients in borough B, are there any significant clinical/social differences (with the potential to affect care required, and therefore cost) seen between the two groups?’

## Method

The method of investigation was a retrospective, in-depth review of patient notes. Notes were selected for investigation from three different clusters within three different London boroughs (served by different mental health trusts). The clusters chosen were two non-psychotic clusters (clusters 5 and 8) and one psychotic cluster (cluster 13). These clusters were selected as likely to contain patients with more complex needs.

The three boroughs were:

- borough A: an inner-city, deprived borough, scoring highly on multiple deprivation indices
- borough B: an outer-London borough also scoring highly on many deprivation indices, but slightly lower than borough A
- borough C: an outer-London affluent borough with low scores on nearly all deprivation indices.

The sample of notes to be reviewed was obtained via the list of patients allocated to each cluster between January and August 2012 within each borough. The start date for the sample was chosen as it was hoped that clustering after 31 December 2011 (national deadline for initial cluster allocation) would be of greater accuracy. The entire list of patients within each borough allocated to the particular cluster at some point in the proposed timeframe was initially provided by the trusts. This list included a mixture of first assessments and cluster reviews, and covered all services provided by the trust. From the original list, a random sample of notes was identified for analysis using a random number generator. The exception to this was where the number of patients allocated to a particular cluster within a borough was low (*n*<50), in which case all the relevant notes were reviewed.

The investigator carried out an initial review of the notes to judge whether the patient had been accurately clustered, as only appropriately clustered patients were to be included. The decision regarding accuracy was made taking into account ‘must score’ items, clinical presentation and history, any previous cluster and the rationale given by the allocating clinician.^6^

For those correctly clustered, the notes were subsequently reviewed in greater detail to collect data on possible complexity factors. This review involved all relevant documentation to enable data collection, including assessments, progress notes and other documents. The data collected covered the following areas: demographic information, MHCT item scores, diagnoses, substance use, risk issues, cultural factors and social factors. Data collected were all from the time of clustering.

### Statistical analysis

Statistical analysis was undertaken with available tools in Excel and using a publicly available spreadsheet.^7^ Owing to

**Fig 1:**
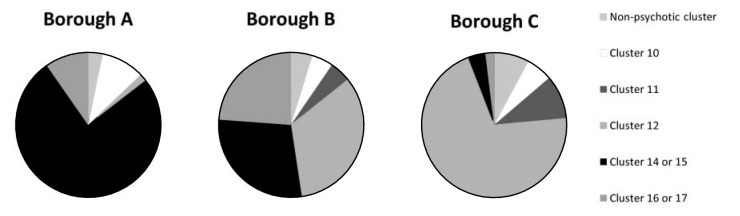
Mental health clustering tool reallocation of cluster 13 (psychotic) patients.

the lower numbers of patients in the cluster 5 and 8 groups, these clusters were analysed together as a non-psychotic group. Where individual scores were collected for each patient on a particular measure, the groups’ mean scores were compared using a two-tailed *t*-test that assumed unequal variance. For category measures, confidence intervals for percentages of patients within particular categories were calculated and then compared for the presence of any statistically significant differences between the groups (significance level set at 95%).

## Results

### Accuracy

A total of 546 patient notes were initially reviewed. Of these, nearly 50% were found to have been allocated an inappropriate cluster, therefore only 283 were included in the ‘deep dive’ analysis.

To provide some analysis of the excluded patients, a decision was made by the investigator as to which cluster would have been more appropriate for these patients based on available information. Trends across the areas were then examined. For the non-psychotic clusters, no area-specific patterns could be seen. However, for the psychotic cluster (cluster 13), a marked area-specific pattern of inaccuracy was seen (Fig. 1). The charts in Fig. 1 show the more appropriate clusters for patients inaccurately allocated to cluster 13, by borough.

It can be seen that in borough A, the inner-city deprived borough, the majority of inaccurately clustered patients would have been more appropriately allocated cluster 14 (or 15), which are the crisis clusters. The setting of clustering in these cases was predominantly on admission to hospital, often under a section of the Mental Health Act. In borough C, the majority of inaccurately clustered patients should have been allocated cluster 12, as these were usually stable community patients with a psychotic illness of medium severity. Borough B showed a more mixed picture.

### Deep-dive analysis

#### Demographics

No significant differences were seen between the areas in terms of cluster gender and age profiles. Ethnic background reflected the population from which the sample was drawn: borough A patients were primarily of White or Black background; the greatest diversity was seen in borough B (including a significant proportion of patients of Asian background); and borough C patients were predominantly of White background.

### Complexity factors

Individual item scores of the MHCT were compared across the areas as illustrated in online Table DS1. The item scores for comparison were chosen as possible markers for complexity, with items reflecting core symptoms and/or ‘must score’ items not included for comparison. The mean area group scores were compared using a *t*-test.

Table DS1 shows that in total 25 out of 72 comparisons (35%) yielded statistically significant differences between the areas. Some of the themes which emerged are discussed below.

#### Risk

Different forms of risk are explored using multiple items of the MHCT, including item 1, item 2, item A, item B, item C, item D and item E. There was a trend towards higher scores for borough B patients on the risk-related MHCT items scores as compared with the other two boroughs. This is particularly evident with the risk to others items (items 1 and A).

#### Substance misuse

It can be seen that for the non-psychotic group, both boroughs A and B patients scored significantly higher on item 3 than borough C patients. There were no significant differences seen between the areas on item 3 scores for the psychotic group, which may reflect the fact that there is a specific cluster available for patients with comorbid substance misuse in this supercluster (cluster 16).

Where patients were using a substance (scoring =1 on item 3), further information was collected from the notes on the type of substance being used. Table 1 shows the breakdown by area of the proportions of each subtype of substance use within this population (all clusters).

It can be seen that there are statistically significant differences between the proportions of patients using alcohol only and multiple substances between the areas. Borough C had a significantly lower proportion of patients with polysubstance use than the other two boroughs (zero in the entire sample), with the majority of borough C

**Table 1:** Substance misuse in the study population in three London boroughs

	% (95% CI)			
Substance	Borough A	Borough B	Borough C	Comparison, *P*
Alcohol only	25 (13-42)	48 (33-63)	68 (52-81)	A < C *P*<0.01
				B = C
				A < B *P*<0.05
				
One drug only	22 (11-39)	15 (7-29)	13 (6-27)	A = C
				B = C
				A = B
				
Two substances (two drugs or alcohol plus one drug)	22 (11-39)	10 (4-23)	18 (9-33)	A = C
				B = C
				A = B
				
Polysubstance	31 (18-49)	28 (16-43)	0 (0-9)	A > C *P*<0.01
				B > C *P*<0.01
				A = B

patients who used any substance falling into the alcohol-only category.

#### Cognition and physical health comorbidity

Table DS1 shows that there were no differences in cognitive function for the non-psychotic group (item 4), however, the psychotic group patients from borough B had greater impairment than patients from boroughs A and C. For the physical health item (item 5), again there were no significant differences seen between the areas for the non-psychotic subgroup. For the psychotic subgroup, borough B and C patients had significantly higher scores than borough A patients. Further data were collected on physical health via the number of physical health diagnoses recorded per patient in the notes, and analysis of these numbers showed that borough C patients had a significantly greater number of diagnoses recorded than patients from boroughs A and B (all clusters).

#### Relationships

It can be seen that for item 9, borough B patients with psychosis scored higher than patients in the other two boroughs, suggesting that these patients had greater difficulty in this area.

#### Activity and occupation

For activities of daily living (item 10), borough B patients with psychosis had greater difficulty in this area than patients in the other two boroughs, with no differences being seen for the non-psychotic group. In terms of occupation, there was an inconsistent picture, with borough C patients in the non-psychotic group scoring higher than borough A patients, and borough B patients in the psychotic group scoring higher than borough C patients. Analysis of employment status documented in the notes showed that borough A patients were most likely to be unemployed, however, the differences between the boroughs were not statistically significant (employment was rare in all boroughs).

#### Accommodation

No differences were seen between the areas on accommodation needs as scored by item 11 for either of the groups. Analysis of accommodation type recorded in the notes showed that borough B had the greatest number of patients who were either of no fixed abode or in supported accommodation, but again the difference between the boroughs was not statistically significant.

#### Other factors

Information was collected on psychiatric comorbidity recording in the notes and statistically significant differences were seen between the areas for the psychotic group patients (borough C having greater comorbidity recorded), but not for the non-psychotic group patients. An attempt was made to examine cultural needs, however this information was difficult to ascertain from the notes. Borough B had a greater proportion of patients who were born overseas as compared with borough C (significant difference), yet by itself this information is of limited value.

Two factors did not show any difference between the different areas: primary diagnosis within cluster and involvement with safeguarding procedures, either safeguarding adult or child protection.

## Discussion

### Limitations

This project was undertaken as part of a leadership fellowship post working with London Health Programmes and Central and North West London NHS Foundation Trust. It was completed by a single investigator without access to statistical software, which limited the possible scale of the investigation and meant that statistical analysis had to be undertaken via available tools in Excel. There were also low total numbers of patients allocated to non-psychotic clusters in some areas, particularly borough A, which further limited the sample sizes.

Attempts were made to limit selection bias by making the procedure for identification of the sample in each area the same. However, those in the borough A sample were significantly more likely to have been in-patients at the time of cluster allocation than those in the borough B and C samples (the vast majority of whom were in the community). This was the case for all the clusters, and therefore presumably represented the fact that at the time of data collection clustering practice in this particular trust was more developed in in-patient than in community settings. This may have affected the comparability of the samples.

In terms of data acquisition, this was straightforward when it came to data from the MHCT, but obtaining information from the notes was more complicated. The boroughs were all served by different mental health trusts who used different IT systems, and some of these systems were easier to extract data from than others. For the additional data collected, this may have had an impact on their quality.

Data quality was found to be a general issue when undertaking this project, both in terms of cluster allocation and potentially in terms of the scoring of individual MHCT items. The process of excluding large numbers of patients from the analysis theoretically could introduce bias, but this is less likely when the more appropriate clusters allocated to the inaccurately clustered patients are taken into account (showing that for the psychotic group the more severely ill patients were included from borough C and the less severely ill patients from borough A).

### Challenges ahead

The results of this project suggest that there may well be clinically and/or socially important differences between patients who have been allocated the same cluster within different areas. About 35% of the comparisons between mean MHCT item scores in different areas were statistically significantly different, with borough B, the outer London deprived borough, showing the greatest level of complexity overall. Although a certain number of positive results would be expected by chance with 72 comparisons being made (typically 5%), this is a far higher percentage than would be expected and cannot be disregarded. Supporting evidence for differences between the areas also comes from some of the additional information acquired from the notes (types of substances used, comorbidity recording).

The findings regarding the accuracy of clustering are also important for a number of reasons. First, a vastly improved accuracy rate than 50% will be necessary for PbR to work in practice. Second, the pattern of inaccuracy elicited can be seen as providing supporting evidence for greater complexity in some geographical areas. The most deprived borough (A) was found to be more likely to ‘under-cluster’ patients with psychosis and the most affluent borough (C) was more likely to ‘over-cluster’ these patients. If this relationship between area deprivation level and coding thresholds were to be consistently reproduced, then it would have significant implications in terms of compounding the possibility of service underfunding in deprived areas. The challenge of benchmarking clinician scores across different areas and different service settings remains significant, and has implications for both training and audit.

#### Tariff implications

Given that there are only 20 clusters available for allocation for all patients receiving mental healthcare, it is to be expected that there will be variation with any given cluster. Variation in itself does not necessarily pose a problem in terms of funding systems if it is consistent across areas. However, if the variation is systematically different in different areas, then an unmodified national tariff would be bound to affect service provision in areas with more complex clinical and social needs. Providers may become reluctant to provide care in these areas or the standard of care may reduce. Whether or not there is a national tariff introduced in mental health, both commissioners and providers will need to be confident that complexity is being captured within a PbR system to ensure that the needs of the local population are fairly represented and to enable agreement on local prices.

### Recommendations

Future studies will be needed to address the complexity question. This project suggests that individual items of the MHCT may play a role in the process of assessing complexity; however, it is unlikely that this will be sufficient and further methods of complexity assessment will need to be developed. An important next step should be an investigation of the relationship of certain complexity factors to the cost of actual care provided. It is likely that a combination of approaches will be needed, including large-scale analysis of readily available data (e.g. MHCT item scores, accommodation/employment status) and smaller prospective studies mapping fewer complexity factors with actual service provision and cost of care. Only after these investigations are undertaken will it be possible to make meaningful decisions about how to proceed with mental health PbR in terms of payment and potential tariff adjustments. There is an understandable reluctance to avoid a system as complicated as that used in acute care, and having as simple a system as possible is a commendable aim; however, this must not be at the expense of adequate resource provision for those with the greatest need.
